# Gene expression patterns associated with blood-feeding in the malaria mosquito *Anopheles gambiae*

**DOI:** 10.1186/1471-2164-6-5

**Published:** 2005-01-14

**Authors:** Ali N Dana, Young S Hong, Marcia K Kern, Maureen E Hillenmeyer, Brent W Harker, Neil F Lobo, James R Hogan, Patricia Romans, Frank H Collins

**Affiliations:** 1 Center for Tropical Disease Research and Training, Department of Biological Sciences, University of Notre Dame, Notre Dame, IN 46556, USA; 2 Department of Zoology, University of Toronto, Toronto, ON M5S 3G5, Canada; 3 Department of Tropical Medicine, Tulane University, New Orleans, LA 70112, USA

## Abstract

**Background:**

Blood feeding, or hematophagy, is a behavior exhibited by female mosquitoes required both for reproduction and for transmission of pathogens. We determined the expression patterns of 3,068 ESTs, representing ~2,000 unique gene transcripts using cDNA microarrays in adult female *Anopheles gambiae *at selected times during the first two days following blood ingestion, at 5 and 30 min during a 40 minute blood meal and at 0, 1, 3, 5, 12, 16, 24 and 48 hours after completion of the blood meal and compared their expression to transcript levels in mosquitoes with access only to a sugar solution.

**Results:**

In blood-fed mosquitoes, 413 unique transcripts, approximately 25% of the total, were expressed at least two-fold above or below their levels in the sugar-fed mosquitoes, at one or more time points. These differentially expressed gene products were clustered using *k*-means clustering into Early Genes, Middle Genes, and Late Genes, containing 144, 130, and 139 unique transcripts, respectively. Several genes from each group were analyzed by quantitative real-time PCR in order to validate the microarray results.

**Conclusion:**

The expression patterns and annotation of the genes in these three groups (Early, Middle, and Late genes) are discussed in the context of female mosquitoes' physiological responses to blood feeding, including blood digestion, peritrophic matrix formation, egg development, and immunity.

## Background

Hematophagy, blood-feeding, is a behavior exhibited by most arthropod vectors of human pathogens. In anautogenous mosquitoes, the female generally feeds to repletion on a single blood meal and then proceeds to use this nutrition as the basis for the development of a batch of eggs. The cycle of host seeking, blood feeding, egg development, and oviposition is generally called the gonotrophic cycle, a term coined by Beklemishev in 1940 [1]. For most mosquitoes living in optimal field or laboratory conditions, this cycle requires about forty-eight hours and involves a complex series of biological events, including peritrophic matrix formation, blood digestion, oocyte development, vitellogenesis, and excretion.

Digestion of the proteinaceous blood meal is required for oocyte development and vitellogenesis, and consequently these are coordinated processes. Multiple hormones interact to alter tissue states and to activate genes involved in these processes. The two hormones juvenile hormone (JH) and 20-hydroxyecdysone (20-E) are most fundamental to ovarian development. Within several days after emergence of female mosquitoes from the puparium, juvenile hormone (JH) stimulates the separation of ovarian follicles from germaria and limited growth of the ovarian follicle to its pre-vitellogenic resting state [2]. JH also confers competence to fat body cells and ovarian follicles for uptake of ecdysteroidogenic hormone (OEH). Then, in response to a blood meal, gonadotrophins are released from cerebral neurosecretory cells and cause the ovaries to become OEH-responsive [3]. OEH stimulates the ovaries to secrete ecdysone, the precursor to 20-E, as well as 20-E during vitellogenesis [4-6]. Fat body cells take up ecdysone, convert it to 20-E and use it to activate transcription of vitellogenin genes [7], the genes encoding the major egg-yolk proteins, as well as a large number of other genes, many of whose products will be incorporated into eggs [see [8,9] for reviews].

Prior to the blood meal, female mosquitoes access sugars for nutritional sustenance. During the first several hours following a blood meal, the mosquito undergoes physiological changes in addition to hormonal ones. Acquisition of a blood meal stimulates midgut proteolytic activity such that approximately 80% of the protein content is digested within one day [10-14]. Serine proteases including trypsins and chymotrypsins are responsible for the majority of endoproteolytic activity [11,12,15]. The role of trypsins in blood digestion has been well documented in *Aedes aegypti*, and more recently it has been investigated in *An. gambiae*. Despite the digestive proteolysis peak at 24 hours post blood meal, digestive enzymes exhibit two phases of transcription [16,17]. In *Ae. aegypti *there are three trypsins, early trypsin, which is constitutively expressed prior to blood feeding and two late trypsins which are blood induced. These two types of trypsins are also found in *An. gambiae*. The *An. gambiae *trypsin family includes seven genes clustered within 11 kb on chromosome 3R, in division 30A, that encode five functional proteins [18]. Trypsins 1 and 2 are both induced by a blood meal and exhibit similar expression profiles. In contrast to Trypsins 1 and 2, Trypsins 3, 4, and 7 are constitutively expressed in unfed females [18]. Trypsins 3 and 7 are down-regulated following a blood meal and not expressed again at levels detectable by RT-PCR until 28 hours post blood meal [18]. In addition to the trypsins, three chymotrypsin genes have been isolated and characterized in *An. gambiae*, two of which are located in tandem on chromosome 2L, in division 25D [19,20]. Both of these genes, AnChym 1 and 2, are expressed in the midgut by 12 hours post blood meal and their transcripts are abundant until 48 hours, as determined by PCR, unlike the levels of Trypsins 1 and 2 that have decreased dramatically by this time [19]. In contrast, the other characterized chymotrypsin, AgChyL, exhibits transcript level changes more similar to those of Trypsins 3–7 [20].

Two types of exopeptidases, carboxypeptidases and aminopeptidases, have been characterized in Anopheline mosquitoes. Edwards et al. [21] cloned a carboxypeptidase that was rapidly induced in *An. gambiae *midguts following blood meal ingestion. Multiple aminopeptidases have been isolated from hematophagous insects, and it has been suggested that they may play different roles in digestion [22-25]. Additional enzymes including glycosidases and lipases are also required for the digestion of non-proteinaceous blood constituents [26,27].

In addition to dramatic changes in physiology, blood feeding also induces changes in mosquito morphology. Following gut distension by blood ingestion, midgut epithelial cells secrete a Type I peritrophic matrix (PM) that is continuous along the length of the midgut [14,28,29]. Prior to the blood meal, the midgut epithelial cells contain high concentrations of apically located, morphologically granular, secretory vesicles. Presumably these apical granules contain precursors of the peritrophic matrix: as early as an hour after the adult female has taken a blood meal, they are no longer detectable [Staubli et al., 1966, as cited in [30]]. In *An. gambiae*, the PM can be visualized by electron microscopy as early as 12 hours PBM and it is fully formed by 48 hours PBM [28,31]. The PM is a biochemically complex structure containing not only chitin and other proteoglycans, but as many as 20–40 different proteins [31-33]. However, only one gene encoding a peritrophic matrix protein has been cloned in *An. gambiae *[34]. The exact functions of the PM remain unknown, but it has been suggested that this semi-permeable porous structure may function as a restrictive layer protecting the midgut epithelium from proteolytic digestive enzymes, from haematin crystals that form following hemoglobin breakdown and as a barrier to blood-borne pathogens including bacteria and malaria parasites [reviewed in [35]].

Once the adult mosquito acquires a blood meal, she spends approximately 48 hours converting about 20% of it into egg constituents [36], using another fraction of it to support the intense biosynthetic activities of this period and defecating the rest. Oogenesis in the mosquito ovary actually begins post-eclosion but oocyte growth attenuates at a resting stage until blood meal ingestion. Once reinitiated, egg development continues until oviposition. Successful egg production not only requires ovarian events for development and maturation of oocytes, but also synthesis of yolk constituents, both protein and lipid, in the fat body, followed by their uptake by oocytes and storage for later use during embryogenesis. Collectively, the events of yolk synthesis, uptake and storage constitute the process of vitellogenesis [30]. Vitellogenesis and oogenesis require the coordination of molecular events in at least these two different abdominal tissues, the fat body and ovary. Based on morphological and physiological criteria, the ovarian cycle can be divided into four phases: 1) Pre-vitellogenic, 2) Initiation, 3) Trophic, and 4) Post-trophic Phase [30].

The meroistic ovary of *An. gambiae *contains approximately 50 functional egg-production structures, the ovarioles. Each ovariole is comprised of two parts, a distal germarium and a vitellarium proximal to a common oviduct through which eggs will pass as they are laid. In the germarium, mitosis of the primordial germ cells creates a syncitium with an oocyte and seven nurse cells interconnected by intracellular bridges, or ring canals as a result of incomplete cytokinesis. Both the germ cell and the nurse cells are surrounded by a somatically derived follicular epithelium [37-39]. The first pre-vitellogenic phase is completed within three days of eclosion and ends with the separation of these follicles from the germaria and entry into the vitellaria. At the end of this phase, oocytes may have undergone some growth but then arrest until events initiated by acquisition of a blood meal cause them to become competent for ovarian vitellogenic events.

Ingestion of a blood meal reinitiates ovarian development and follicle growth resumes. In *Ae. aegypti *and *Anopheles albimanus*, this period appears variable, lasting 3–10 and 8–16 hours, respectively, and ends with the initiation of vitellogenin synthesis [30]. In the next two days, during the trophic phase, the mosquito generates large amounts of vitellogenin, the secreted precursor to the major yolk protein vitellin. In addition to vitellogenin, the developing oocytes also accumulate other proteins, and lipids from the hemolymph, as well as ribosomes and mRNAs synthesized in the syncitial nurse cells. These latter constituents are transported to the germ cell through the ring canals connecting the oocytes and nurse cells by a process of cytoplasmic streaming [40]. Following delivery, several maternal mRNAs become localized within the oocyte. These maternal transcripts are fundamental for dorsal/ventral and anterior/posterior patterning of the embryo that will develop from the oocyte. This pattern of deposition and the patterning of the eggshell also depend on a complex signaling process involving both the somatic cells of the follicular epithelium and the oocyte.

Once oocyte growth has ceased, vitellogenin synthesis terminates. This signals the onset of the post-trophic phase. During this time, the oocytes mature and eggshell structures begin to develop. The chorion, part of the eggshell, is secreted by the follicular epithelium and contains two layers, the first secreted, inner endochorion and the later secreted, outer exochorion [30]. It is the endochorionic layer that will harden and melanize after oviposition.

Specialization of eggshell structures necessitates communication between cells. The RAS 1 signaling cascade is an important means of communication during the processes of oocyte and eggshell patterning, as it is during eye development and differentiation of structures late in embryogenesis [41,42]. During patterning in *Drosophila*, developing oocytes produce the TGFα protein Gurken that binds to the epidermal growth factor receptor (EGFR), a receptor tyrosine kinase (RTK) localized to the posterior follicle cells, to initiate RAS 1 signaling. Downstream from the activation of this RTK, the GTP-binding protein RAS 1 initiates a series of enzymatic events propagated successively by three protein kinases, RAF, MAPK, and MAPK kinase (MEK), resulting in the translocation of nuclear factors and possibly the concomitant reorganization of the cytoskeleton [reviewed in [43]]. Thus, a cascade of events leads to the establishment of the posterior follicle cell fate. The posterior follicle cells then signal back to the germ cells. This results in the reorganization of the oocyte cytoskeleton, and regulates the localization of anterior/posterior determinants. Similar to the eggshell, the oocyte also undergoes dorsal-ventral patterning. Following patterning of the follicle cells, maternal gene products are regulated by the Toll signaling pathway to generate a transcription factor gradient that will spatially regulate activity of specific zygotic genes within the fertilized oocytes [44].

Vitellogenic events in the fat body have also been divided into phases: 1) Pre-vitellogenic, 2) Vitellogenic, and 3) Termination. The pre-vitellogenic phase in the fat body coincides with the pre-vitellogenic phase of the ovarian cycle. During this phase, RNA synthesis increases in the fat body and the rough endoplasmic reticulum and the Golgi complex proliferate to prepare for the production of vitellogenin. At the start of the vitellogenic phase, the release of mosquito hormones initiated by digestion signal the onset of vitellogenesis [45]. Synthesis of large amounts of vitellogenins is facilitated by the large quantities of biosynthetic machinery generated during pre-vitellogenic stages, but also depends on the presence of multiple vitellogenin genes (Romans, unpublished). Following synthesis, vitellogenin is released into the hemolymph and eventually diffuses through channels between the cells of the follicular epithelium, whereupon it is accumulated by the oocytes by a process of receptor-mediated endocytosis in clathrin-coated pits [8]. When vitellogenesis has ceased, during the termination phase, the biosynthetic machinery in the fat body is degraded via a lysosomal pathway, at least in *Ae. aegypti *[46].

Thus, blood feeding initiates a complex series of physiological events in at least three tissues that are integrated by the actions of JH, 20-E and peptide hormones. These events may be required for parasite development; they certainly can be modulated by the presence of parasites [47,48] and may provide points of intervention for mosquito control. Microarray analysis provides a tool to study global expression patterns of thousands of genes simultaneously. By comparing the level of transcription of a gene over time between two states, *e.g*. blood-fed *vs*. sugar-fed, an expression signature for each gene can be defined in response to blood feeding. Consequently, these expression patterns may indicate how these genes are regulated and interact, and also the biological processes in which the act. In this study we performed microarray analysis of genes in female mosquito abdomens during the first 48 hours after a blood meal. We have implicated many of these genes in different processes stimulated *de novo *by blood feeding. The elucidation of the expression profiles of abdominal genes will provide a broadened basis for understanding vector-parasite interactions. Our study certainly provides insights into the physiology of the malaria vector *Anopheles gambiae*.

## Results

### Array composition

Microarray analysis was conducted on 3057 cDNA clones generated from three different adult female *An. gambiae *mosquito abdomen-derived cDNA libraries to elucidate major patterns of gene expression through 48 hours post ingestion of a blood meal. Arrays were constructed from triplicate spotted negative controls (purified water, 3 × SSC with no DNA, and empty wells), positive controls for blood-fed samples consisting of 3 clones whose ESTs corresponded to rat (*R. norvegicus*) α and β hemoglobin chains, and PCR-amplified fragments obtained from 1132, 721, and 1204 clones randomly picked from the sugar-fed (harvested after 30 hours at 19°C), rat blood-fed (harvested 30 hours PBM at 19°C), and *P. berghei *infected rat blood-fed (harvested 30 hours PBM at 19°C) abdomen libraries, respectively (Table 1). Approximately 84% of PCR-amplified fragments were visualized on ethidium bromide stained 1% agarose, 1 × TBE gels prior to spotting (data not shown). Of these PCR-amplified fragments, 2219 clones (87% of electrophoresed PCR products) were represented by a single defined band (Table 2).

**Table 1:** Microarray composition

***Controls***	**Clones**
**Negative Controls**	**108**
**Positive Controls**	**3**

**Libraries**	

Sugar-fed Adult Female (incubated 30 hours at 19°C) Abdomen library	1132
Blood-fed Adult Female (incubated 30 hours PBM at 19°C) Abdomen library	721
*Plasmodium berghei *Blood-fed Adult Female (incubated 30 hours PBM at 19°C) Abdomen library	1204

Total	3168

**Table 2:** Appearance of PCR product following gel electrophoresis

	**Total Clones**	**Singlet**	**Doublet**	**Smear**	**No Product Visible**
Amplification of 2558 Clones	2558	2098 (82%)	117 (5%)	6 (<1%)	337 (13%)
Re-amplification of 183 Clones previously amplified with "No Product Visible"	183	121 (66%)	14 (8%)	0	48 (26%)

Cumulative	2558	2219 (87%)	131 (5%)	6 (<1%)	202 (8%)

Note: PCR-amplified fragments were visualized on ethidium bromide stained 1% agarose, 1 × TBE gels. Approximately 13% of PCR products could not be visualized following the first amplification due to product yield below the threshold of ethidium bromide detection; 54% of these (183) were re-amplified.

ESTs corresponding to these spotted cDNAs were screened for mitochondrial contamination, filtered based on sequence trace file quality, and assembled (EST clustered) using the DNAstar Seqman II software (DNAstar, CA) (Table 3). The high quality ESTs clustered into 491 contigs (consensus sequence generated from ≥2 overlapping ESTs) and 1415 singletons (ESTs with no sequence similarity to any other EST in the assembly) for a total of 1906 unique transcripts (Table 4).

**Table 3:** EST composition of array

	**Number**	**Percentage**
High Quality Sequence Data	2707	88.56%
Poor Quality Sequence Data	131	4.28%
Mitochondrial DNA	222	7.25%

Total	3060	100%

Note: EST analysis includes the ESTs of the positive controls (*Rattus norvegicus *hemoglobin chains).

**Table 4:** Putative transcripts represented on the array following EST assembly

	**Number**	**ESTs Represented**
Contigs	491	1292
Singletons	1415	1415

Total	1906	2707

Note: EST assembly includes the ESTs of the positive controls (*Rattus norvegicus *hemoglobin chains).

### Microarray and bioinformatic analyses

Global patterns of greater than two-fold up-regulation or down-regulation for these cDNAs were established by comparing transcript levels in blood-fed *An. gambiae *adult females at ten time points during and post ingestion of a blood meal to the levels in sugar-fed females. First strand cDNA was generated from total RNA collected at 5 min and 30 min after initiation of blood feeding and at 0, 1, 3, 5, 12, 24, and 48 hr post-blood meal. All cDNA populations were labeled and hybridized to arrays. For each PCR-amplified insert, Cy3 and Cy5 fluorescent dye levels were measured from 3 replicate spots on each of 50 arrays to generate average signal intensities, and an expression ratio depicting transcript fold change between sugar-fed and blood-fed mosquitoes calculated. Following quality control filtering and normalization, 456 cDNAs and the rat β-hemoglobin gene, the positive control, were expressed more than twofold above or below control, sugar-fed levels at one or more of the 10 blood-feeding time points. Following EST analysis, the 456 cDNAs were found to represent 413 unique mosquito transcripts, 10 of which were present in more than one set. This anomaly is due to EST clustering of alternatively spliced transcripts with different expression patterns. More unique transcripts are up-regulated than down-regulated in response to blood feeding, while 10% of them are both up-regulated and down-regulated over the time course of this study: 192 are up-regulated at least twofold, 173 are down-regulated at least twofold, and 48 are down-regulated and up-regulated. Bioinformatic analyses of these 413 unique transcripts showed that all sequences shared sequence identity with the *An. gambiae *genome (Table 5), 90% of which shared sequence identity with an entry in Nr of dbEST (Table 5). In this analysis Blast hits with an E value ≤1 × 10^-4 ^were considered significant.

**Table 5:** Sequence similarity of consensus sequences

	**Blastn (WGS *An. gambiae*)**	**Blastx (Nr)**	**Blastn (Nr)**	**Blastn (dbEST)**	**Unannotated**	**Total**
3 Positive Controls	No Significant Hit	3				3

403 Unique Transcripts	403	240	8	112	43	403

Early Genes	144	77	2	45	20	144
Middle Genes	130	78	6	36	10	130
Late Genes	139	93	2	31	13	139

Total	413	248	10	112	43	413

### Microarray gene clustering and principal components analysis

**Figure 1:**
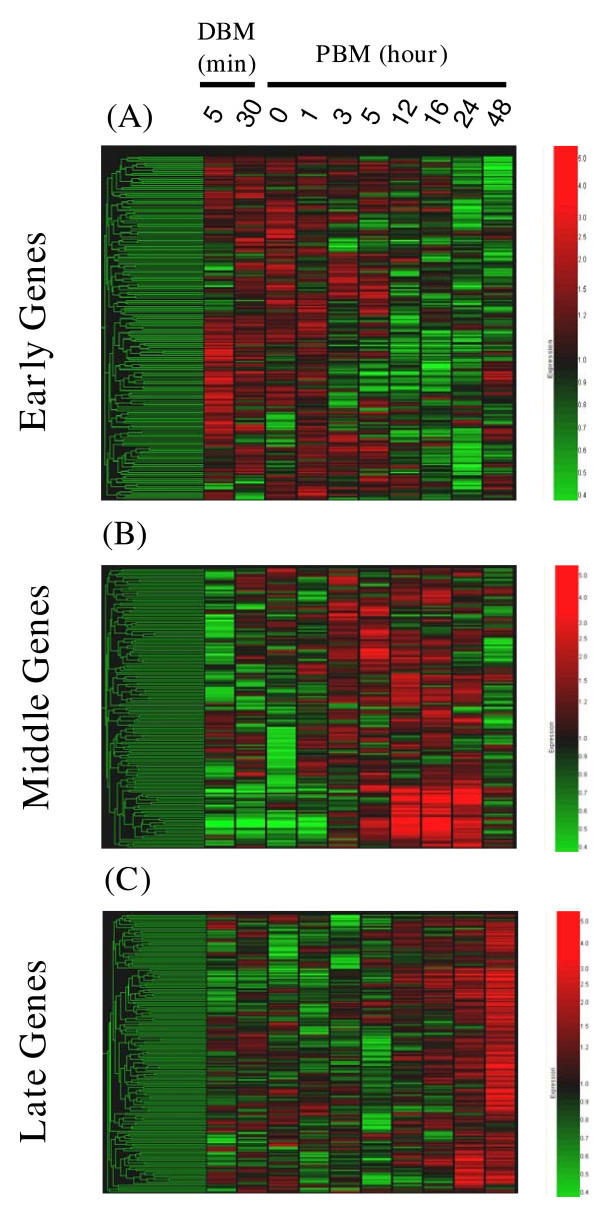
Gene trees displaying the microarray generated expression profiles of abdomen-derived cDNAs in blood-fed compared to sugar-fed adult female mosquitoes during the following times: 5 and 30 minutes during blood meal (DBM), 0, 1, 3, 5, 12, 16, 24, and 48 hours post blood meal (PBM). *k*-means clustering of all genes up-regulated and down-regulated at least two-fold during at least one of the ten time points generated three sets of genes. These *k*-means-derived groups of genes were hierarchically clustered for visualization and include Set 1 designated the Early Genes (A), Set 2 designated the Middle Genes (B), and Set 3 designated the Late Genes (C). Each gene is represented by a single row of colored boxes; each time point is represented as a single column of colored boxes. The expression scale is represented as a gradation of color ranging from 5 fold induced genes indicated by saturated red to 2.5 fold repressed genes indicated by saturated green.

The behaviors of the gene products identified as at least twofold up/down-regulated were grouped into three sets using *k*-means clustering (Figure 1) and named according to the time of their induction during the 48-hour time course following the initiation of blood feeding. Set 1, hereafter referred to as the "Early Genes", contains 144 unique transcripts derived from 152 cDNAs, which are expressed mainly during the early time points (Table 6, Figure 1A). The majority of these genes are appear induced at least twofold more abundantly than in sugar-fed mosquitoes during the first five minutes of blood feeding. Many of these transcripts remain induced until 1-hour PBM, although some remain induced until 5 hours PBM. After 5 hours post blood meal, the majority of Early Genes is down-regulated and they remain down-regulated even 48 hours after blood meal ingestion. A small subset of the Early Genes shows a variant pattern of gene expression in which the transcripts are up-regulated from the first 5 minutes of blood uptake through 1 hour PBM followed by a repression in expression from 3 to 24 hours PBM and then a greater than twofold induction at 48 hours PBM. The 130 unique transcripts represented by 147 cDNAs in Set 2, the "Middle Genes", follow a more dynamic pattern of gene expression than the Early Genes (Table 6 and Figure 1B). Most Middle Genes are down-regulated in blood-fed versus sugar-fed mosquitoes until 3 hours PBM followed by an increase in expression commencing at 5 hours PBM and peaking between 12 and 24 hours PBM. Subsequently, Middle Genes are down-regulated to initial transcript abundances by 48 hours PBM. Also, in a behavior largely exhibited by the Middle Genes, approximately 40% of genes are down-regulated when the mosquitoes completed feeding and left the rat (0 hours PBM). Set 3, the "Late Genes" contains 139 unique transcripts, 157 cDNAs, which are either down-regulated or constitutively expressed until 12 to 16 hours PBM after which they are up-regulated and, in contrast to the Middle Genes, continue to be highly expressed even at 48 hours PBM (Table 6, Figure 1C).

**Table 6:** Bioinformatic analysis of two-fold expressed genes

	**Early Genes**	**Middle Genes**	**Late Genes**	**Total**
Twofold Expressed cDNAs (represented by consensus sequences >100 bp in length)	152	147	157	456
Replicate Consensus Sequences within *k*-means Sets	8	17	18	43
Unique Transcripts	144	130	139	403*
Unique Transcripts included within PCA Analysis	82	69	98	249

*The complete data set (Early, Middle and Late genes combined) contains 413 unique transcripts. However, ten transcripts are present in two different gene sets and were counted twice as a result.

**Figure 2:**
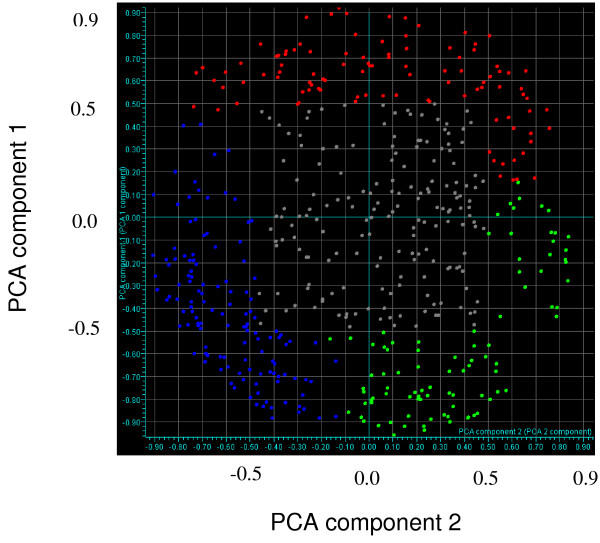
Scatter plot of the first two components for each gene that is either up-regulated or down-regulated at least two-fold during one time point in the course of this experiment. The Early Genes, Middle Genes, and Late Genes are colored red, blue and green, respectively. The genes colored in grey include Early, Middle and Late genes that did not have a value greater than 0.5 or less than -0.5 of both PCA 1 and PCA 2 components.

**Figure 3:**
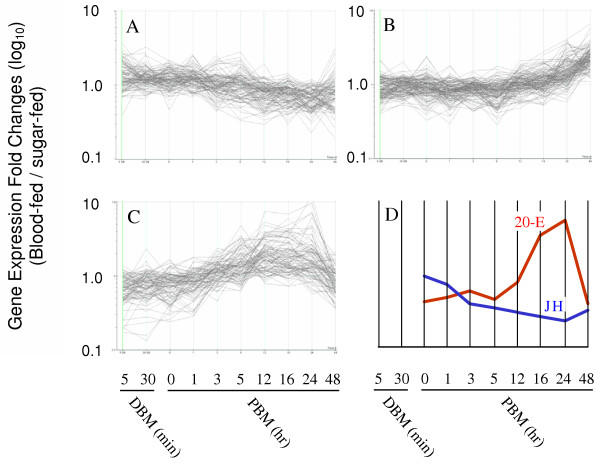
Parallel coordinates display of expression profiles of differentially expressed PCA-filtered genes from the Early Genes (A), Middle Genes (B), and Late Genes (C). X-axes correspond to a successive time point. Y-axes denote the ratio of fluorescent intensities of blood-fed to sugar-fed samples at each time point for each gene in Panels (A), (B), and (C). Plotted genes had a PCA 1 value greater than 0.5 or less than -0.5, or a PCA 2 value greater than 0.5 or less than -0.5. In Panel (D), the activity levels of juvenile hormone (JH) and ecdysone (20-E) are plotted on a similar parallel coordinate graph (modified from Dhadialla and Raikhel 1994).

Principal components analysis (PCA) of these 413 unique differentially expressed transcripts was also conducted to support the *k*-means defined sets. Following PCA, each transcript was plotted in a scatter plot comparing the PCA 1 and PCA 2 values. The three *k*-means-defined sets of genes did not overlap on these scatter plots. Thus the PCA results support classification of these transcripts into three groups. For each of the *k*-means defined groups, genes that had a PCA 1 or PCA 2 value greater than 0.5 or less than -0.5 were plotted on a parallel coordinates display (Figures 2 and 3). The resulting data sets contained 82, 69, and 98 unique transcripts representing Early Genes, Middle Genes, and Late Genes, respectively (Table 6).

### Gene annotation and gene ontology assignments

To identify *An. gambiae *genes whose products are involved in related processes, the EST consensus sequences of the transcripts differentially expressed in these 3 patterns were annotated using sequence similarity and categorized using the molecular functions listed by the Gene Ontology Consortium (GOC) and the biological processes defined by Holt et al. [49]. Gene annotations for all 413 at least twofold differentially expressed gene products are given in the Supplementary Table S1. They were then categorized into 9 major categories with 31 subdivisions (Table 7). 48% of the genes could not be annotated and therefore were categorized as "Unknown". The three most numerous categories containing annotated gene products were "Metabolism", "Protein Synthesis", and "Egg Production".

**Table 7:** Functional annotation of AS represented by microarray expression group

	**Early**	**Middle**	**Late**
**Metabolism**			

Simple/Complex Carbohydrate Metabolism and Transport	3	0	2
Oxidative Phosphorylation	8	5	1
Lysosomal Enzymatic Digestion	0	0	2
Protein Digestion	4	4	3
Protein Modification, Metabolism, Transport and Localization	5	12	8
Amino Acid and Derivative Metabolism and Transport	1	8	2
Nucleobase/Nucleoside/Nucleotide/Nucleic acid Metabolism and Transport	4	1	3
Fatty Acid/Lipid Metabolism and Transport	1	0	2
Vitamin/Vitamin Derivative/Cofactor Metabolism and Transport	2	0	0
Xenobiotic Metabolism and Transport	1	0	2

Total	29	30	25

**Transport**			

Ion Transport	3	3	0
Receptor-mediated Endocytosis	1	0	2

Total	4	3	2

**Protein Synthesis**			

Transcription and mRNA Processing	2	5	6
Translation	11	10	4
Protein Folding	3	4	4

Total	16	19	14

**Cellular Processes**			

Cell Cycle	0	3	3
Cellular Proliferation	0	0	2
Chromatin Assembly/Disassembly	0	0	5
Apoptosis	1	0	0
Senescence	1	0	0

Total	2	3	10

**Egg Production**			

Vitellogenesis/Oogenesis/Embryogenesis	2	10	7
Melanization	0	1	0

Total	2	11	7

**Cellular Communication**			

Signal Transduction	1	1	2
Cell-cell Signaling	3	0	1

Total	4	1	3

**Intra-/Extra-cellular Architecture Maintenance**			

Structural	3	4	2
Muscle-related	1	0	0
Cell Adhesion	1	1	0
Cytoskeleton Organization and Biogenesis	1	0	2

Total	6	5	4

**Response to Stress/External Stimulus**			

Response to Oxidative Stress	2	1	3
Immune/Defense Response	2	2	3

Total	4	3	6

**Unknown**			

Total	77	55	68

During the 48 hours PBM, the majority of gene products involved in metabolism were up-regulated Early and Middle Genes. Largely different metabolic biological processes were up-regulated in Early vs. Middle and Late Genes. More than half of the Early metabolic gene products, 20/29 unique transcripts, appear to be involved in carbohydrate metabolism, oxidative phosphorylation, and protein digestion. In contrast, 80% of the metabolic genes, 24/30 unique transcripts, represented in the Middle Genes contribute to various processes in protein digestion and metabolism, and metabolism of amino acids and their derivatives. One third of the Late genes involved in metabolism, 8/25 unique transcripts, are involved in protein metabolism. Five of these annotated sequences, ASs 368, 807, 1179, 279, and 922, encode products involved in post-translational modification.

Reflecting the necessity of biosynthetic machinery in cell maintenance and growth, and probably also the highly conserved nature of proteins involved in housekeeping functions, the protein synthesis category contained the second largest number of genes functioning in a known process. 69%, 11/16 unique transcripts and 53%, 10/19 unique transcripts, respectively of the Early and Middle Genes, in this category are involved in translation. Approximately 25%, 5/19 unique transcripts, and 40%, 6/14 unique transcripts, of the protein synthesis genes represented among Middle and Late genes, are required for transcription and mRNA processing. This result seems almost paradoxical because transcription and mRNA processing necessarily precede translation.

A number of biological processes were related by their involvement in nuclear events or the overall activity of the cell. There were three times as many genes involved in cellular processes among the Late genes than in the other two sets combined. Although all the genes involved in the cell cycle are Middle and Late Genes, the most strikingly up-regulated cellular process genes were those involved in chromatin assembly/disassembly. 5 of the 10 cellular process Late genes (ASs 1136, 59, 592, 1011, and 1792) are involved in maintenance of chromatin structure, a biological process represented only in the Late genes. These Late genes include both histones and high mobility group proteins.

The majority of significantly up and down regulated genes appear to function in egg production, either in the development and maturation of oocytes or in the fat body synthesis of products that will be deposited in oocytes. Almost 90% of them, 17/19 unique transcripts, are Middle and Late genes. These genes are described in detail in the Discussion section. In contrast, half of the genes categorized as cellular communication genes, 4/8 unique transcripts, are Early genes. The majority of the cellular communication gene products in the combined sets of Middle and Late genes, are involved in different signal transduction pathways. Additionally, almost half of the intra-/extracellular architecture maintenance genes are Early genes. This category includes a wide variety of gene products such as peritrophin, both muscle-related and cytoskeletal actins, α-catenin and β-integrin. The Middle and Late genes in this category were mainly structural and included two peritrophins (ASs 13 and 642).

The biological process categorized as transport included not only the movement of ions such as zinc, sodium and potassium, but also transport of molecules via receptor-mediated endocytosis. All three transport gene products, ASs 1336, 1605, and 432, in the Middle genes are responsible for the movement of ions. In contrast, in the Early and Late genes, several gene products (ASs 1974, 1071, and 2086) appear to be involved in receptor-mediated transport via clathrin-coated vesicles.

A number of genes responding to oxidative stress (6 genes in total) were found in all three sets of genes indicating that they are transcribed throughout the 48 hours PBM. Seven additional gene products most probably involved in immunity, a response to external stress, were found among these three gene sets.

### qRT-PCR analysis

**Figure 4:**
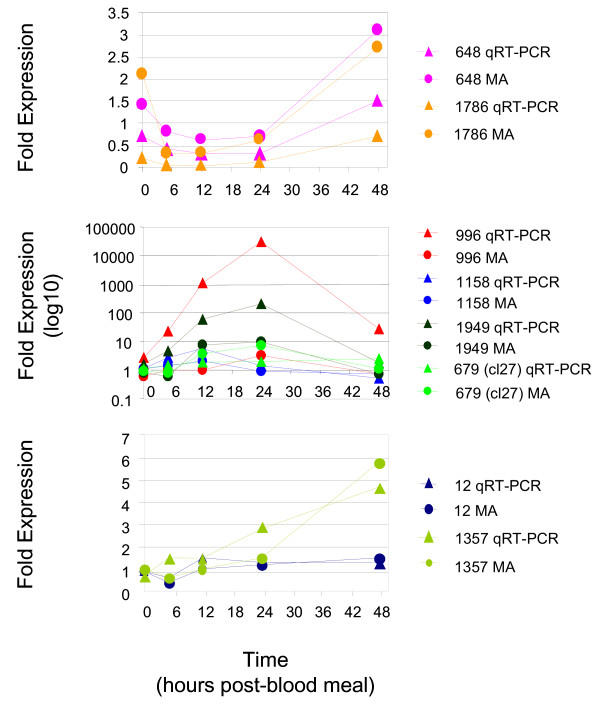
Comparison of microarray and qRT-PCR gene expression profiles for selected genes. X-Y plots were generated from the ratio of transcript levels in the blood-fed adult female mosquitoes to the transcript levels in the sugar-fed adult female mosquitoes for eight selected genes at five time points including 0, 5, 12, 24 and 48 hours post blood meal (PBM). Genes were randomly selected from the three sets and included ASs 648 and 1786 from the Early Genes (A), ASs 996, 1158, 1949, and 679 from the Middle Genes (B), and ASs 12 and 1357 from the Late Genes (C). Triangles indicate fold expression data generated by qRT-PCR analysis; circles indicate fold expression data generated by microarray analysis. A horizontal line connecting either the diamonds or circles illustrates each gene expression profile. For each gene, the expression profiles created by qRT-PCR and microarray analysis are indicated using the same color.

Expression profiles of eight selected genes and the RP S7 control gene were confirmed using a quantitative real-time PCR strategy (Figure 4). Transcript levels for each of the eight genes were quantified using SYBR Green technology and differences in their expression between sugar-fed and blood-fed mosquitoes at 0, 5, 12, 24 and 48 hours PBM determined. Although the magnitudes of the changes in transcript abundances of all the genes whose expression levels were quantified by both techniques differed between the techniques, the changes in direction of expression, whether positive or negative, remained consistent for the majority of them. In addition, the overall patterns of expression exhibited by the three sets of genes were also apparent in the expression profiles created by qRT-PCR analysis. For the two Early genes, microarray analysis overestimated transcript levels between 2- and 30-fold more than qRT-PCR analysis. In contrast, for the majority of the Middle and Late gene expression measurements, microarray analysis underestimated transcript abundances relative to qRT-PCR analysis.

## Discussion

We have determined the gene expression patterns of 3,068 abdomen-derived cDNAs from adult female *An. gambiae *mosquitoes representing 1906 unique transcripts were determined in the first two days following ingestion of a blood meal by microarray analysis. 413 unique transcripts were shown to be up-or down-regulated at least twofold in blood fed mosquitoes relative to sugar-fed mosquitoes at one or more of the following times: 5 min and 30 min following initiation of blood feeding and 0, 1, 3, 5, 12, 24, and 48 hr post-blood meal. These transcripts were clustered into three sets with different temporal patterns of expression that may reflect the major hormonal changes occurring within the mosquito during a gonotrophic cycle. These differentially expressed gene products were annotated putatively using sequence similarity searches and categorized by biological process to identify the major events occurring post blood meal ingestion in the female mosquito.

Multiple hormones interact to alter tissue states and to activate genes involved in the female mosquito's digestion of a blood meal, in oocyte development and in vitellogenesis. The three sets of differentially transcribed genes discerned in this study, the Early, Middle and Late genes may reflect differential hormonal responsiveness. After acquisition of a blood meal, the transcript levels of the Early genes which were abundant during blood feeding showed general declines until 24 hours PBM, after which a subset of transcript levels began to rise again. Expression of Early genes may be linked to the relatively high titers of JH present at the beginning of the first gonotrophic cycle and may then be repressed as a result of declining JH titers or of increasing 20-E. The expression of Middle gene transcripts followed an expression pattern that reflects the titers of 20-E: levels sharply increased by 12 hours PBM, remained stable or increased only slightly until 24 hours PBM, and then declined rapidly. OEH secreted by median neurosecretory cells stimulates the ovaries to secrete 20-E during vitellogenesis and the activity of this hormone begins to rise by 3–5 hours, peaks between 12 and 24 hours, and then declines to baseline levels by 48 hours post blood ingestion [4-6]. In contrast to the Middle genes, most Late gene transcripts exhibited baseline, steady state levels until 12 hours PBM after which they were induced at least twofold and continued to exhibit increased transcript levels at 48 hours PBM. This increase in transcript levels mirrored the increase in JH titer observed by 48 hours post blood meal ingestion [50]. These results suggest that Middle genes products may be ecdysone-responsive whereas Late genes products may be JH-responsive.

Based on their finding that the *Drosophila minidiscs *gene product showed a primary response to JH, Dubrovsky et al. [51] have suggested that JH may transcriptionally regulate genes encoding maternally inherited products. Our Late gene, AS 806, shares sequence similarity with *minidiscs*. Additionally, the majority of mosquito gene products showing sequence similarity to maternally active *Drosophila *genes are categorized as Late genes. Whether transcription of Early genes is directly influenced by levels of JH or 20-E, cannot be determined easily because neither hormone is present at high levels during the first few hours following blood meal ingestion. Regardless of expression pattern, the gene products in each set reflect a diverse array of processes occurring in the female mosquito within 48 hours following initiation of blood feeding. The major processes initiated in response to blood feeding including digestion, peritrophic matrix formation, oogenesis and vitellogenesis, are discussed below with emphasis on the likely roles of particular gene products.

### Digestion

Digestion of the two different food sources, nectar sugars and blood, requires changes in the types of enzymes present within the digestive tract of the mosquito. The numbers of genes associated with sugar and protein metabolism within each set of genes may reflect the switch from sugar to protein metabolism. For instance, there are increases in transcript abundance of genes involved in carbohydrate metabolism and oxidative phosphorylation in the Early genes. However, both Early and Middle gene categories are enriched in genes involved in protein digestion. This result stems from the involvement of certain gene products in blood meal digestion that initiate a signaling cascade resulting in the up-regulation of other related proteolytic enzymes. The Middle genes contain the majority of gene products involved in amino acid metabolism, a process that follows protein digestion, whereas by 48 hours PBM, the time at which the majority of Late genes are induced, there is a generalized decrease in digestive enzyme transcripts.

Considering that blood contains large quantities of protein, the mosquito requires a variety of proteolytic enzymes to digest the recently acquired meal. In the present study, 11 genes were identified whose products are most likely required for protein digestion. These include 5 previously characterized digestive enzyme genes, two trypsins, a chymotrypsin, a serine protease and a carboxypeptidase. The majority of these digestive enzyme genes were transcribed at levels greater than twofold induction after 6 hours PBM. The *An. gambiae *Trypsins 1 and 2 are both induced by a blood meal and exhibit similar expression profiles although Trypsin 1 is expressed at higher levels. Muller and coworkers, using an RT-PCR strategy, showed that Trypsin 2 mRNA is present at 8, 12, 24 and 28 hours post blood meal [18,52]. In our study, the Trypsin 2 gene (AS 569) also exhibited increased transcript abundance at 12 hours PBM with maximal expression occurring at 24 hours PBM, but these levels decreased by 48 hours. In contrast to Trypsins 1 and 2, Trypsins 3, 4, and 7 are constitutively expressed in non-bloodfed females. By 4 hours following a blood meal, levels of Trypsin 4 become undetectable by Northern and RT-PCR analysis and do not reappear until 20 hours PBM [18] and unlike Trypsins 1 and 2, Trypsin 4 reaches maximal expression by 48 hours, near the end of the gonotrophic cycle. Our study identified two clones corresponding to Trypsin 4 (AS 568) but the two cDNAs exhibited different expression patterns. One (Accession no. CD747033) reached maximal transcript abundance at 48 hours PBM, the expected expression pattern. However, the other cDNA (Accession no. CD747029) was expressed at high levels prior to 6 hours PBM and also reached at least twofold increased levels by 48 hours PBM. These cDNAs may not have clustered together for technical reasons intrinsic to Seqman II, or they may correspond to alternatively spliced variants of the same gene. The sequence alignment showed 97% nucleotide sequence identity between the two ESTs over the region common to both. However, the CD747033 EST is only 253 bp in length and it is possible that the full-length cDNA represents an alternatively spliced transcript. In addition to Trypsins 2 and 4, we identified a trypsin-like serine protease (AS 648) which shared the greatest amino acid similarity with Trypsin 4. However, this serine protease exhibited highest nucleotide identity to a different region of chromosome 3R than that containing all previously identified digestive trypsins. This gene product was induced greater than twofold within 6 hours PBM and was repressed during the height of digestion. Unlike Trypsins 3–7, it was not expressed at higher levels at 48 hours PBM. It is possible that this trypsin is not involved in digestion but in another proteolytic process that is down-regulated following a blood meal.

Barillas-Mury *et al*. [53] demonstrated that the early trypsin activity is essential to the transcription and subsequent expression of the late trypsins in *Ae. aegypti*. *An. gambiae *Trypsins 3–7 may indirectly activate transcription and increase the expression of Trypsins 1 and 2, the major endoproteolytic enzymes required for blood meal digestion [18]. In both *Ae. aegypti *and *An. gambiae*, these early expressed digestive enzymes are presumed to act as signal transducers causing transcriptional up-regulation of the late expressed ones [14,53]. Thus the digestive process is regulated by an elaborate biphasic expression pattern of serine proteases. Additional evidence suggests that not only tryptic peptides but cleaved amino acids may serve as systemic signals regulating subsequent processes. In contrast to the trypsin by-products, cleaved amino acids may also function as negative regulators of food intake. AS 1158 shared weak sequence similarity with the *Drosophila pumpless *protein, a larval fat body-expressed enzyme involved in glycine catabolism. In the fruit fly, larvae expressing the *pumpless *mutation are unable to pump food from the pharynx to the esophagus [54]. These mutant animals do not feed, neither do they upregulate genes normally involved in responses to starvation. Because feeding amino acids to wild type larvae phenocopied effects of the *pumpless *mutation, Zinke *et al*. [54] proposed that amino acids released from the fat body normally act as signals for cessation of feeding.

In addition to the trypsins, three chymotrypsin genes have been characterized in *An. gambiae *[19,20]. The expression of two of these digestive enzymes, AnChym 1 and 2, has been localized to the mosquito midgut by analysis of Northern blots [19]. RT-PCR showed that both chymotrypsin genes are expressed at 12 hours PBM and are abundant until 48 hours PBM, unlike transcripts of Trypsins 1 and 2 which have decreased dramatically by this time [19]. The cDNA representing chymotrypsin 2, AS 99, was categorized as a Late gene since maximal transcript levels were achieved after 24 hours PBM. In contrast, the other characterized chymotrypsin, AgChyL, exhibits changes in transcript abundance which is more similar to those of Trypsins 3–7. mRNA is present in non-blood fed females, detectable until 8 hours post blood meal after which mRNA can no longer be measured until 48 hours PBM [20]. The cDNA corresponding to this chymotrypsin-like serine protease, AS 994, was induced more than twofold prior to the peak of digestion and clustered with the Early Genes. In addition to the aforementioned chymotrypsins, our study identified a previously uncharacterized chymotrypsin (AS 2243) also located on chromosome 2L but in a different region from both AnChym1 and AnChym2. Exhibiting an expression pattern different from both AnChym2 and AgChyL, this gene product was characterized as a Middle Gene with maximal transcript levels achieved between 12 and 24 hours PBM and a return to baseline by 48 hours PBM, similar to the expression patterns of Trypsins 1 and 2.

Edwards *et al*. [21] investigated expression levels of *An. gambiae *carboxypeptidase A following blood meal ingestion. Northern blot analysis indicated that levels of Carboxypeptidase A mRNA rose rapidly to a ten-fold increase within 3–4 hours following a blood meal, then dropped to the pre-induction state by 24 hours PBM. We identified a carboxypeptidase gene located on chromosome 2L (AS 1742) that is transcribed in a manner similar to that of carboxypeptidase A. However another cDNA representing a carboxypeptidase (AS 44) exhibited a radically different expression pattern. Transcripts were present at low levels 1–5 hours PBM but increased more than twofold between 12 and 24 hours PBM, a pattern that resembled the enzymatic activity in *An. stephensi *observed by Jahan *et al*. [13], namely a rapid increase until 12 hours PBM, with a peak at 24 hours, followed by a steady decline over the next day.

In contrast to the *An. gambiae *carboxypeptidase A, the levels of aminopeptidase peaked around 30 hours in *An. stephensi *[12]. Additionally, Lemos *et al*. [14] recorded peak aminopeptidase activity at 24 hours PBM in *An. gambiae*. We identified two aminopeptidases with at least two-fold increased expression during the 48 hours PBM. The first aminopeptidase, AS 340, reached peak transcript levels at 24 hours PBM, showing a similar expression pattern to the enzyme activity levels reported by Billingsley and Hecker [12] and Lemos *et al*. [14]. In contrast, the other aminopeptidase, AS 430, showed amino acid similarity to the *Ae. aegypti *aminopeptidase N. This aminopeptidase was classified as a Late gene, due to increased transcript levels at 24 hours PBM but maximal levels were not achieved until 48 hours PBM. Jahan *et al*. [13] documented two different kinetic profiles of aminopeptidase enzymes in *An. stephensi *depending on whether the enzyme was soluble or membrane-associated. The soluble aminopeptidase exhibited a kinetic profile similar to AS 340 and to that presented by Billingsley and Hecker [12] with peak activity at 24 hours PBM.

Geering [55] had suggested that phospholipase activity plays a role in blood digestion in *Ae. aegypti *although no conclusive evidence was demonstrated. However, Geering and Freyvogel [56] demonstrated that lipolytic activity increased 15 hours after blood feeding. Of the three gene products that are characterized as being involved in Fatty Acid/Lipid Metabolism and Transport, two, ASs 1177 and 997, encoding an acetate-CoA ligase and a fatty acid binding molecule, respectively, are expressed at more than twofold greater abundance at 24 hours PBM. These gene products may be involved in fatty acid degradation of blood meal components and the transport of these lipids to the oocytes.

The erythrocyte membrane contains a number of glycoproteins. It is therefore possible that enzymes normally associated with carbohydrate metabolism of nectar meals could also be involved in blood digestion. Almost all of the Simple/Complex Carbohydrate Metabolism and Transport genes identified in our study as being at least twofold upregulated were categorized as Early genes. Within the first 6 hours PBM, their transcripts are present at higher levels than in sugar-fed females and thereafter, they decrease steadily until 12 and 24 hours PBM. This expression profile does not exclude these genes from having a role in RBC glycoprotein metabolism. Several glycosidases are present within the midgut of *An. stephensi*, either associated with the lumen or with epithelial lysosomes [12]. The enzymatic activity of α-glucosidase, the major midgut glycosidase in *An. stephensi*, increased from 6 hours PBM to maximal levels by 24 hours and decreased to basal levels by 36 hours PBM in the anterior midgut. The transcript abundance of the α-glucosidase, AS1786, characterized in this study followed a different pattern than the enzymatic activity of *An. stephensi *α-glucosidase. It showed greatest amino acid sequence similarity to *Drosophila melanogaster *gene product CG8690 α-glucosidase and was categorized as an Early Gene with minimal transcript levels occurring at and after 24 hours PBM.

### Peritrophic matrix formation

Shen and Jacobs-Lorena [34] characterized the *An. gambiae *peritrophic matrix protein Peritrophin 1 (Ag-Aper1) by analysis of Northern blots, and demonstrated that transcripts were present 6 hours PBM, increased by 12 hours, and remained elevated between 24–48 hours PBM. The present study identified several genes encoding proteins with a chitin-binding domain (InterPro ID IPR002557: Chitin binding Peritrophin-A) similar to the one found in Peritrophin 1. The Early gene AS 928 contains the Peritrophin-A chitin binding domain and maps to chromosome 3L *in silico*, corresponding to agCP10685. Another Early gene, AS 13, shows high identity to Peritrophin 1. A Middle gene, AS 516, is expressed by 6 hours PBM but does not reach maximal levels until 24 hours PBM. This gene product maps *in silico *to chromosome 2L, exhibits a similar transcript profile, and also shares 98% amino acid identity with Peritrophin 1. It is not clear why two sets of ESTs, both identified as Peritrophin 1, should exhibit different transcription patterns unless they are derived from differentially regulated genes. Another Middle gene, AS 1164, contains the Peritrophin-A chitin binding domain in addition to a prenyl-group binding site (InterPro ID IPR001230: CAAX box). This Middle gene may not be involved in peritrophic matrix formation but in some other process coinciding with protein digestion. Two other cDNAs, Accessions CD746211 and CD746202, both in AS 13, could also be localized to chromosome 2L. However, their ESTs exhibited greatest nucleotide identity to the gene predicted as ENSANGG00000020776 located 4 kb 3' to Ag-Aper1. These two cDNAs clustered as Early and Late genes respectively, and may represent alternatively spliced gene products.

As early as an hour after the adult female has taken a blood meal, secretory vesicles previously present in the apical brush border of midgut epithelial cells are no longer detectable [Staubli et al., 1966 as cited in [30]]. These apical granules presumably contain precursors of the peritrophic matrix. The Middle and Late peritrophin gene products may be packaged into vesicles in preparation for a subsequent blood meal. In contrast, the Early peritrophin genes may be transcribed in response to blood meal acquisition and their products used immediately in the formation of the peritrophic matrix.

### Ovarian cycle and oogenesis

The extensive literature on genes involved in *Drosophila *ovarian development and early embryogenesis opens windows into interpreting our *An. gambiae *microarray results and understanding mosquito egg development. The majority of *An. gambiae *genes upregulated at least twofold following a blood meal appear to function in egg production. The only gene possibly involved in oogenesis during the early phases of the ovarian cycle is the Early gene AS 670. It shares sequence similarity with *peter pan*, a *Drosophila *gene product required during oogenesis. Oocytes in *peter pan *mutants often have an incorrect number of associated nurse cells, suggesting that the *peter pan *protein influences the separation of cells within the germaria [57]. The identification of other genes involved in the early stages of mosquito oogenesis may be facilitated by the construction of cDNA libraries from the abdomens or ovaries of recently blood fed female mosquitoes.

Several differentially expressed gene products found in the present study may be involved in the formation of ring canals, structures necessary for the delivery of maternal factors to oocytes. In particular, bulk transfer of cytoplasmic content from nurse cells to oocytes depends on actin structures [40]. A Middle gene product, AS 679, shares sequence similarity to the *Drosophila *gene CG13388 encoding the protein kinase anchor protein 200, *Akap200*. *Akap200 *protein localizes to ring canals during oogenesis, regulates protein kinase C activity, and controls their morphology [58]. Late gene product AS 1317 shows sequence similarity to the *Drosophila pendulin *gene product, encoded by CG4799. This gene product is also required for assembly of fully functional ring canals. *pendulin *encodes an importin-α2, a protein necessary for the localization of the *kelch *gene product, CG7210. *kelch *encodes an actin organizer without which the ring canals become occluded and nurse cell-oocyte cytoplasmic transport is inhibited [59,60]. Though we found an apparent *pendulin *gene, we did not find *kelch*. A Late gene product, AS 578, is the homolog of *Cdc42*, which encodes a small monomeric RHO GTPase involved in signal transduction. Rohatgi *et al*. [61] suggested that *Cdc42 *protein most likely links signal transduction to the actin cytoskeleton in *Xenopus*. In *Drosophila *ovaries, mutations in *Cdc42 *caused nurse cells to deflate and coalesce, and inhibited transfer of nurse cell cytoplasm to oocytes in late stage egg chambers [62].

*Drosophila *nurse cells transcribe the *bicoid *anterior determinant gene and the resulting mRNA is transported to the anterior region of developing oocytes via polarized microtubules [63]. *bicoid *does not appear to be an anterior determinant in other insects, but other genes important for its localization are conserved. The Early gene AS 2047 shares similarity with the *Drosophila cornichon *gene, CG5855. In *Drosophila*, *cornichon *is required for formation of a functional microtubular cytoskeletal scaffold used to transport *bicoid *mRNA and the posterior group *oskar *gene product to their proper location within the embryo [64]. The Late gene product, AS 1044 exhibits greatest sequence similarity to *D. virilis exuperantia*, a gene whose product is also required for proper *bicoid *mRNA localization *D. melanogaster *[65,66]. The Late gene AS 2222 is putatively identified as the *An. gambiae *homolog of *Drosophila Notch*. In *Drosophila*, *Notch *signaling regulates a large number of ovarian events beginning with cyst development in the germarium and extending through oogenesis [67]. The mechanisms by which *Notch *signaling activates transcription of its target genes are reviewed by Barolo and Posakony [68]. Since we identified *Notch *as a Late gene, its activities may be more restricted in *An. gambiae*, and/or reflect fundamental differences in ovarian biology. AS 1391, a Late gene product, shares sequence similarity with *Drosophila Rab-protein 11*. This *Drosophila *small monomeric RAB GTPase is also involved in the polarization of the microtubules for the organization of the posterior pole and for *oskar *localization there [69].

To regulate the progress of oogenesis and embryogenesis, stored maternal mRNAs are translationally repressed during early oocyte development. The Middle gene product AS 2031 shares sequence similarity with the *Drosophila *gene product *Bicaudal C*, a RNA binding protein that may play a role in translational silencing of maternal mRNAs in addition to its role in eggshell patterning [70]. Mutations in *Bicaudal C *result in premature translation of *oskar *mRNA before it has reached the posterior region of the oocyte [71]. The Middle gene product AS 1490 is the putative homolog for the *Drosophila *gene product *vasa *(CG3506), an ATP dependent helicase involved in pole plasm assembly that may also be involved in translational modification of maternal mRNAs [72]. The Late gene AS 453 shares sequence similarity with the *Drosophila cup *protein (CG11181). *cup *protein interacts with *nanos*, the posterior determinant, and a translational regulator of the gap gene *hunchback *mRNA during oogenesis, although the exact function of the *cup *protein still remains unknown [73]. A DEAD box protein encoded by *vasa *also influences oocyte differentiation and the development of the *Drosophila *embryo body plan via translation of *oskar*, *nanos*, and g*urken *during oogenesis [74-77]. In amphibians, several mRNA binding proteins have been identified that are only present in oocytes and not post cleavage embryos [78,79]. One *An. gambiae *Middle gene product, AS 2449, shared sequence similarity with the *Xenopus laevis *poly(A)-specific ribonuclease and also has mRNA binding motifs, thus it may also repress translation of mRNAs in embryos.

Maternal nurse cells not only provide the biosynthetic machinery and mRNA needed for oocyte axis determination, but also many transcripts and proteins required for zygotic development through the cellular blastoderm stage. The Middle gene product AS 1032 shares sequence similarity with *nop5*, encoding a maternally derived product of the *Drosophila *CG10206 gene, a component of the small nucleolar ribonucleoprotein (snoRNP) complex involved in rRNA processing [80]. The Late gene product AS 806, referred to above in the context of its possible regulation by JH, shares sequence similarity with the *Drosophila minidiscs *gene product, an amino acid transporter. In *Drosophila *ovarian nurse cells, JH induces the expression of *minidisks *and its transcripts are most likely transferred to the oocyte during nurse cell cytoplasmic streaming [51].

Similar to the oocyte, the eggshell undergoes dorsal-ventral patterning. Crucial to this process is the correct placement of the oocyte relative to the maternal somatic follicle cells. In *Drosophila*, the localization of the oocyte depends on cadherin-associated adhesion [81]. The Late gene product AS 1890 is the homolog of α-catenin, the CG17947 gene product. A cytoskeletal anchor protein, α-catenin is required for positioning of the oocyte relative to the posterior follicle cells during germ cell rearrangement in *Drosophila *[81]. The RAS 1 signaling cascade is an important means of cell communication during embryo and eggshell patterning [41,42]. The Middle gene product AS 657 is weakly similar to the *Drosophila Star *protein, a RAS 1 enhancer involved in the EGF receptor signaling pathway, either upstream or in parallel to EGFR, during formation of the embryonic ventral midline. *Star *encodes a single pass transmembrane protein that may be involved in the processing of *gurken *protein. The DNA damage checkpoint *14-3-3epsilon *protein also participates in RAS 1 signaling, normally functioning downstream or in parallel to RAF, but upstream of transcription factors. The Middle gene AS 106 shares sequence similarity with *Drosophila 14-3-3epsilon*. The *14-3-3epsilon *protein is also capable of binding to a large number of other proteins in a phosphorylation-dependent manner. One of its functions may be to alter the cell cycle by binding Cyclin B and appears to have homologs in most if not all eukaryotes [82,83]. The *An. gambiae Cyclin B *homolog, AS 1357, grouped as a Late gene.

Mutation screens in *Drosophila *have led to the identification of a number of other gene products that may be involved in RAS 1 signaling. *TppII (tripeptidyl-peptidase II)*, and *smt3 *(SUMO) were discovered in a search for lethal mutations that could enhance a weak RAS 1 eggshell phenotype [84]. The Middle gene product AS 1268 shares sequence similarity with *Drosophila tripeptidyl-peptidase II*, the CG3991 gene, encoding a serine protease that degrades neuropeptide signals [85]. The Late gene product AS 922 is the homolog of *Drosophila smt3*, CG4494, whose product is ubiquitin-like protein that may tag proteins for nuclear localization or retention in the cytoplasm [86,87]. *smt3 *protein may modulate activity of transcription factors in the follicle cells downstream of EGFR activation. However, we feel that *smt3 *is likely to be a minor player in RAS 1 signaling in the events following blood ingestion in the mosquito, because its mRNA reaches maximal expression after 12–24 hours PBM, unlike the other gene products we identified as potentially influencing RAS 1 signaling.

*smt3 *protein may also play a role in *Toll *signaling. This signal transduction pathway is known to be necessary for dorsal/ventral patterning of the *Drosophila *embryo. *smt3 *protein binds the NFκB homolog *dorsal *protein and targets this Rel transcription factor for migration to the nucleus [88,89]. Bhaskar *et al*. [88] demonstrated that *smt3 *conjugation to the *dorsal *protein enhanced its transcriptional activity. *smt3 *protein may play other roles in the cell by altering the interactions of septins, cytoskeletal proteins involved in cytokinesis [90,91]. In *Drosophila*, septins have been found in the cytoplasm of nurse cells and at the baso-lateral surfaces of follicle cells [92]. These results suggest even more pleiotrophc roles for the *smt3 *gene product in oogenesis. We also found that the Late gene AS 2034, a homolog of *Drosophila Aos1*, the CG12276 gene, the *smt3 *(SUMO) activating enzyme, was also expressed at least twofold more abundantly during the height of *smt3 *expression. This result reinforces the importance of *smt3 *in the events occurring between 24–48 hours PBM.

In addition to genes regulating the polarity of the embryo and eggshell, genes involved in cellular growth and differentiation were differentially expressed during the 48 hours PBM ingestion. AS 337 and 495 shared sequence similarity with the *Ae. aegypti *ornithine decarboxylase antizyme, a protein that modulates polyamine synthesis. The homologous *Drosophila ornithine decarboxylase antizyme *gene, formerly known as *gut feeling*, has been shown to be important in developing oocytes. It is one target of *Sex lethal *which encodes an RNA binding protein that regulates mRNA splicing and the mitotic events in early germ cells via regulating Cyclin B [93]. The Late gene product AS 2073 shares sequence similarity with the *Drosophila polo *CG12306 gene product, a protein kinase required for cytokinesis and another regulator of Cyclin B [94]. The Early gene AS 1972 shows identity with the *Drosophila black pearl *CG5268 gene product. This protein contains DnaJ domains implying that it is necessary for cellular growth [95]. Northern blot analysis of *black pearl *RNA from various developmental stages showed two transcripts with greatest expression in *Drosophila *embryos 0–6 hours old [95], the stages in which DNA replication recurs most rapidly. The Late gene AS 2268 shares sequence similarity with the *Drosophila Imaginal disc growth factor4 *(*Idgf4*), a mitogen with a non-functional chitinase domain. Transcripts of *Idgf4 *are detected in *Drosophila *nurse cells, oocytes, and in the yolk cytoplasm of early embryos [96]. The Middle gene product AS 2273 shares sequence similarity with *Drosophila β Integrin*. *An. gambiae *β Integrin may interact with the Middle gene AS 985 product to promote somatic cell adhesion and cell migration during oogenesis and embryogenesis. This is due to the similarity of the AS 985 gene to *Drosophila Receptor of activated protein kinase C*, RACK1. RACK1 can bind a number of different signaling and cell adhesion molecules including the activated form of protein kinase C (PKC), Src family kinases, and β Integrins [97-99]. Cox *et al*. [100] demonstrated that, in a mammalian system, RACK1 organizes focal adhesions and directional cell migration via its Src-binding site. Mahairaki *et al*. [101] found that the *An. gambiae *β Integrin gene was expressed at highest levels 48 hours PBM, whereas we found that the β integrin homolog reached at least twofold increased expression by 24 hours PBM.

A number of genes have been implicated in the development of the egg shell structures. Our screen does not appear to have identified any homologs of the several endochorionic structural proteins characterized in *Ae. aegypti *[102,103]. This was unexpected because Northern blot analysis had indicated that transcription of the vitelline membrane proteins 15a-1, 15a-2, 15a-3 was induced rapidly between 10 and 24 hours PBM, reached maximal levels between 30 and 40 hours PBM, and decreased to baseline levels between 50 and 60 hours PBM [102,103]. Our study also did not identify a *Dopa decarboxylase (Ddc) *gene, Ddc is an enzyme involved in the tyrosine metabolic pathway necessary for eventual chorion melanization in *Ae. aegypti*, and other melanization events. The gene is up-regulated in response to blood meal with transcripts initially detectable by 12 hours PBM, and maximal levels achieved between 24 and 48 hours PBM [104]. However, we did identify a gene encoding another enzyme involved in tyrosine metabolism. AS 1340, a Middle gene product, shared sequence similarity with the *Ae*. *aegypti *Dopachrome conversion enzyme [105]. This enzyme is required for processing of dopachrome to melanin. It is interesting that its mRNA is constitutively expressed in *Ae. aegypti *females, but becomes upregulated when they are infected with *Dirofilaria *[105]. Since insect melanins can be produced via any of three intermediates, Dopa, Dopamine, or Dopachrome, it may be that *An. gambiae *differs from *Ae. aegypti *in the substrate metabolized to produce chorionic melanin.

Several Middle and Late genes encoding antioxidants were upregulated at least twofold 12–48 hours PBM. The Middle gene, AS 2033, a glutathione S-transferase D3, and the three Late genes, ASs 1684, 35, and 2156, encoding glutathione S-transferase 1–6 class theta, and homologs of *Drosophila *thioredoxin and *Ae. aegypti *2-Cys thioredoxin peroxidase, may have roles in regulating reactive oxygen species that can be produced from the highly reactive quinones which are normally cross-linked into melanin immediately after they are formed.

### Ovarian cycle and vitellogenesis

Paramount to the development of the embryo is the massive accumulation of vitellogenin by the oocyte. In *An. gambiae *there is a small, polymorphic tandem array of vitellogenin genes and a single dispersed vitellogenin gene, all located on Chromosome 2R in division 18B (P. Romans and M. Sharakhova, unpublished observations). Vitellogenin mRNA becomes detectable by Northern blot analysis by 8 hrs PBM, though it is detectable earlier by RT-PCR, increases dramatically by 12 hours, reaches maximal levels by 24 hours, and declines to undetectable levels by 48 hours PBM [47]. Our microarray study identified three cDNAs, all Middle gene products and greater than twofold induced, as vitellogenin gene homologs. Two of the ESTs were not conjoined during EST assembly because they represented non-overlapping 5' and 3' ends of the Vg1 gene. The third EST included the more closely resembled the sequence of the dispersed vitellogenin gene (P. Romans and A. Dana, unpublished). As expected, all three vitellogenin clones exhibited expression profiles similar to the overall pattern previously described [47].

Following synthesis in the fat body, vitellogenins are released into the hemolymph. Eventually, they diffuse through channels between the cells of the follicular epithelium and are accumulated by the oocyte by receptor-mediated endocytosis in clathrin-coated pits [8]. The increased number of gene products involved in receptor-mediated endocytosis before and after the height of vitellogenin gene transcription, 12–24 hours PBM in this study, may reflect a preparation for the increase in receptor-mediated endocytosis when the oocytes are accumulating vitellogenins and other yolk constituents during the trophic phase of the ovarian cycle. These genes included an Early gene, AS 1974, similar to the *Drosophila *Adaptin subunit, *AP-1σ*, CG5864, and the Late gene, AS 2086, homolog of another *Drosophila *clathrin-associated protein, *AP-50*, CG7057.

When vitellogenesis has ceased, the biosynthetic machinery in the fat body is degraded in lysosomes [46]. In *Ae. aegypti*, the lysosomal cathepsin D-like aspartic protease (AeLAP) exhibited a similar transcription profile to vitellogenin [106]. Cho *et al*. [107] also identified a Cathepsin B-like thiol protease, vitellogenic Cathepsin B or VCB, which is secreted from the fat body with a peak at 24 hours PBM and incorporated into oocytes. It appears to be involved in the degradation of vitellin in embryos. The Middle gene AS 996, a Cathepsin B, shares identity with this *Ae. aegypti *protein, exhibits the same expression profile, and may be its homolog. At approximately 30 hours PBM, 6 hours after peak production of vitellogenin, the activity of four other lysosomal enzymes, arylsulfatase A, acid phosphatase-1, β-galactosidase, and Cathepsin D, has dramatically increased to reach maximal levels by 36–42 hours PBM [108,109]. The two Late gene products, ASs 1254 and 2231, were identified putatively as the lysosomal enzymes, acid phosphatase-1 and Cathepsin F, respectively. These genes also may be involved in the termination phase of vitellogenesis. Cathepsin F is necessary for oocyte growth in a teleost fish and has been suggested to be associated with yolk protein processing [110]. It will be a very interesting example of gene co-evolution, should processing of vitellogenins, proteins conserved between egg-laying vertebrates and non-Brachyceran insects, actually be accomplished by similarly conserved cathepsins.

## Conclusions

Holt *et al*. [49] performed the first genomic-scale study of hematophagy in *An. gambiae *by identifying 168 ESTs that differed in statistical abundance between cDNA libraries made from adult female mosquitoes fed on sugar and 24 hours PBM. Ribeiro [111] extended this study by describing an additional 267 such genes. We have expanded on these studies by identifying additional 359 ESTs and by examining virtually a complete first gonotrophic cycle experimentally. In addition, we found 18 ESTs present in our microarray and Ribeiro's [111] studies. All but one (AS 205) showed the same expression patterns at 24 hours PBM (Table 8). These highly synchronous expression profiles of those ESTs further validate that experimental microarray and *in silico *data can complement each other. However, our study is unique in that we have determined the temporal patterns of expression of the genes we identified. The observed similarities between the gene expression patterns and production of the two principal insect hormones suggest that gene transcription may be influenced by changes in JH titers as well as by 20-E levels, a phenomenon that has been well-studied in the context of *Drosophila *metamorphosis and in *Ae. aegypti *vitellogenesis. Future analysis may reveal genes co-regulated via the same promoters. Indeed, this now appears possible for organisms whose genomes have been sequenced [112-114]. As new regulatory sequences are identified, the arsenal of transcriptional regulators to drive their tissue- and stage-specific gene expression will be increased. We expect that this increased promoter availability will supplement current vector-control strategies.

**Table 8:** List of genes differentially expressed* in female *A. gambiae *at 24 hours post-blood meal in both microarray and *in silico *(Ribeiro, 2003) gene expression studies.

**AS ID**	**Ensembl ID**	**Microarray**	***in silico***	**Molecular Functions**	**Biological Processes**
99	agCP3123	Up	Up	enzyme	Protein Digestion
996	agCP14019	Up	Up	enzyme	Egg Development
2222	agCP8969	Up	Up	unknown	Unknown
1949	agCP12846	Up	Up	unknown	Unknown
1317	agCP8818	Up	Up	transporter	Transport
516	agCP3409	Up	Up	binding	Structural
1044	agCP3927	Up	Up	unknown	Egg Development
995	agCP5701	Up	Up	enzyme	Protein Digestion
230	agCP2518	Up	Up	nutrient reservoir	Egg Development
180	agCP1111	Up	Up	unknown	Unknown
2207	agCP15442	Up	Up	transporter	Ion Transport
2256	agCP2731	Up	Up	unknown	Unknown
2243	agCP3610	Up	Up	enzyme	Protein Digestion
205	agCP5849**	Down	Up	unknown	Unknown
86	agCP6049	Down	Down	unknown	Unknown
553	agCP11425	Down	Down	transporter	Oxidative phosphorylation
2123	agCP11416	Down	Down	transporter	Transport
642	agCP8191	Down	Down	structural molecule	Cuticle biosynthesis

*; These genes displayed at least 2-fold up- or down-regulation relative to the control.

**: AS 205 shows discrepancy between microarray and *in silico *expression data.

Up: up-regulation; Down: down-regulation.

Great progress has been made in the annotation of the *An. gambiae *genome, culminating in the public announcement of the genome sequence in 2002 and its subsequent updates. Yet, although we have identified 413 differentially expressed gene products, we could not assign almost half of them to a biological process. Of the 200 "Unknowns," 43 unique transcripts shared no significant identity with sequences in the Nr and dbEST databases. The genes corresponding to these transcripts may be identified following the second gene build of the *An. gambiae *genome. Functional studies using microarray analysis verified by qRT-PCR must confirm *in silico *predicted annotations and provide biological information about gene products. Many of the gene products identified in this study share sequence similarity with *Drosophila *proteins. Much of the information generated by studies of fruit fly cell biology and development may also apply to mosquitoes, although it will be more difficult to test in *An. gambiae*, since it is not easily manipulated genetically. This study underscores the importance of ongoing functional studies including tissue-specific expression profiling using microarray analysis and qRT-PCR. Understanding how the events following blood feeding are related to each other on a molecular level will provide a more comprehensive picture of this unique behavior and may also delineate new vector-control strategies.

## Methods

### Microarray chip fabrication

Three cDNA libraries were constructed from abdomens of adult female *An. gambiae *which had been sugar-fed (harvested 30 hours post-eclosion), rat blood-fed (harvested 30 hours PBM), and *P. berghei*-infected rat blood-fed (harvested 30 hours PBM), all at 19°C (Dana, unpublished PhD thesis). Clones from all three libraries were subjected to PCR-based insert amplification using λTriplEx2 vector specific primers (3' LD Amplimer Primer 5'-ATACGACTCACTATAGGGCGAATTGGC-3'; 5' LD Amplimer Primer: 5'-CTCGGGAAGCGCGCCATTGTGTTGG-3'). Amplification reactions contained 1.0 μL eluted phage, 0.03 pmol of each primer, 1 × Taq Polymerase Buffer (Invitrogen), 3 mM MgCl_2_, 1 mM of each dNTP, and 0.2 U Taq Polymerase (Invitrogen), in a total volume of 100 μL. Reactions were conducted in 96-well plates on a Perkin-Elmer 9700 Thermocycler using the following cycling conditions: initial denaturation at 95°C for 5 min, followed by 35 cycles of denaturation at 94°C for 30 s, annealing/elongation at 70°C for 2 min, and a final elongation step at 68°C for 3 min. Samples of all PCR products were electrophoresed on 1% agarose, 1 × TBE gels and visualized by ethidium bromide staining. PCR products were purified on a Beckman Biomek FX using Montage PCR 96 Cleanup kits (Millipore), eluted in 100 μL of water, evaporated overnight and the pellets resuspended in 30 μL of 3 × SSC microarray spotting buffer. A total of 3060 resuspended cDNA inserts and 108 controls were spotted in triplicate on CMT-Gaps II slides (Corning, NY) using the Affymetrix Arrayer 417 at 19 – 20°C and relative humidity between 50 – 60%. Slides were post-processed by baking at 80°C for three hours, incubation in 1% SDS for 2 min, in 95°C purified water for a further 2 min, and then plunged 20 times into 100% ethanol kept at -20°C and air-dried via centrifugation at 500 RPM for 5 min.

### Microarray target preparation and hybridization

Total RNA was extracted from blood-fed and sugar-fed whole adult female mosquitoes of the malaria susceptible 4Arr strain, 5–7 days post eclosion, using Trizol (Molecular Research Center, Inc) according to the manufacturer's directions. Mosquitoes were blood-fed on anesthetized white rats and maintained under conditions similar to those for sugar-fed mosquitoes, 25°C with 80% humidity and a 12-h light/dark cycle with available 20% sucrose solution, until collection. Fully-engorged mosquitoes were identified visually and harvested at the following 10 time-points: 1) 5 min after initiation of blood feeding during the acquisition of the blood meal (DBM), 2) 30 min DBM, 3) 0 hr post-blood meal (PBM), immediately after they ceased feeding on the rat, 4) 1 hr PBM, 5) 3 hr PBM, 6) 5 hr PBM, 7) 12 hr PBM, 8) 16 hr PBM, 9) 24 hr PBM, and l0) 48 hr PBM. For each blood-fed sample, total RNA was extracted from batches of approximately 10–15 females. Total RNA was also extracted from batches of 100 sugar-fed females for reference samples. RNA samples were then treated with 1.0 μL DNase I (Life Science Technology) according to manufacturer's instructions. Following DNase I treatment, total RNA was re-extracted with Trizol. First strand cDNA synthesis and labeling with Cyanine 3 (Cy3) or Cyanine 5 (Cy5), were performed on 15 μg of total RNA from each sample using the Genisphere 3DNA Array 50 kit according to the manufacturer's protocol. Hybridizations were conducted following the two step protocol recommended by the manufacturer: 1) cDNA hybridization to the amplified cDNA probes spotted on the slides, 2) hybridization of 3-DNA fluorescent dendrimers (Genisphere) to cDNAs via the capture sequences incorporated into them during first strand synthesis. All cDNA and fluorescent dye hybridizations were performed in a volume of 50 μL using the formamide-based hybridization buffer provided by the manufacturer. The cDNA hybridizations were performed at 45°C overnight. The slides were then washed according to the 3DNA Array 50 kit protocol and air dried by centrifugation for 3 min at 800 RPM. The 3-DNA hybridizations were performed at 53°C for 2 hours as described above, except that 0.5 mM DTT was added to the first two wash solutions to protect the fluorochromes from oxidation. Five replicate slides were generated for each of the ten time points for a total of 50 hybridized and labeled slides. These included two dye-swap experiments performed to eliminate dye fluorescence bias. Pilot experiments conducted with total RNA from the same sample labeled with both Cy3 and Cy5, self-self hybridization, indicated that there was no dye labeling bias following data normalization (data not shown).

### Microarray data acquisition and statistical analysis

Following hybridization and washing, microarray slides were scanned successively at 532 and 635 nm using the Affymetrix 428 Array Scanner. Raw signal intensities were acquired using the adaptive circle algorithm and spot intensities quantified using the Jaguar 2.0 segmentation and data analysis software (Affymetrix, CA). Average signal intensities were normalized using the Loess curve for intensity dependent normalization followed by a per gene median normalization using the Genespring 5.1 software (Silicon Genetics, CA). Signal intensities were filtered such that only gene products exhibiting a raw signal intensity value greater than 300 pixels in one channel and greater than two-fold expression difference between the sugar-fed and blood-fed samples from at least one time point hybridized to the same array were utilized in further analysis. Gene expression level measurements falling outside one standard deviation from the mean signal intensity of each gene product calculated from the five replicates were excluded from further analysis. As an additional quality control, only genes whose PCR amplified products migrated as a single band in agarose gel electrophoresis and that generated high quality sequences for use in EST assembly were analyzed.

Gene products that were induced or repressed at least twofold during blood feeding were initially clustered hierarchically using the Genespring software to determine the user-defined number of centroids (clusters) to be used in *k*-means clustering (data not shown). From this preliminary analysis it was determined that three major clades existed and the genes were clustered using Genespring software using a *k*-means clustering algorithm with a centroid number of 3 and the Pearson Correlation distance metric. Finally, an independent analysis using principal components analysis (PCA) was conducted on the genes induced or repressed at least twofold, using the Genespring software.

### qRT-PCR

Transcript levels of several selected genes were measured using SYBR dye technology (Applied Biosystems, CA) and quantitative real-time PCR (qRT-PCR) analysis in order to validate microarray data,. The Primer Express Software v. 1.5 (Applied Biosystems, CA) was used to design the following primers to nine genes: the two Early Genes agCP4871 (AS 648; Forward 5'-TGATTCGTGCCAGGGTGAT-3'; Reverse 5'-CACCACACCAACAAGGACATC-3') and CG8690 (AS 1786; Forward 5'-GCTGACTTTGAGCGGTTGG-3'; Reverse 5'-CACAAAGTCCATGATCACCTTCA-3'), the four Middle Genes agCP8064 (AS 679; Forward 5'-TGGCGAGGTCGATCAGCTA-3'; Reverse 5'-CATTATCGCCATCGTTGTGTTG-3'), agCP12846 (AS 1949; Forward 5'-TTTGTGGTTCGGTATCGATCTG-3'; Reverse 5'-CGAGCACTTTGGCGAACTTC-3'), CG7758 (AS 1158; Forward 5'-CACGGTTGGCATTTCGAAC-3'; Reverse 5'-GCAGCTGTGCGAACACCA-3'), and agCP14019 (AS 996; Forward 5'-GTCGGGCGATTCCAATGA-3'; Reverse 5'-TGTAACCGGGCTGGCAAA-3'), and the two Late Genes agCP14623 (AS 12; Forward 5'-CGGCAAATCGGTTCAGCT-3'; Reverse 5'-TGAATCGGTGCCTTGCG-3') and agCP2112 (AS 1357; Forward 5'-CCTGCATGAAGGTGGAATGA-3'; Reverse 5'-TTGCCAAGCTCTCCCAACAC-3'), and the ribosomal protein S7 (RP S7) gene control (Forward 5'-CATTCTGCCCAAACCGATG-3'; Reverse 5'-AACGCGGTCTCTTCTGCTTG-3'). RP S7 was used as an internal control since its expression is constitutive during blood-feeding [115-118]. All amplifications and fluorescence quantification were performed using an ABI 7700 Sequence Detection System and associated Sequence Detector Software v. 1.7 (Applied Biosystems, CA). Standard curves were generated using 10-fold serial dilutions of genomic DNA (ranging from 0.0116 to 116 ng per reaction). These qPCR reactions were performed in duplicate in a total volume of 25 μL containing 12.5 μL of SYBR green PCR Master Mix, 300 nmol of each primer, and nuclease free water (Gibco, UltraPURE) using the following conditions; 50°C for 2 min, then denaturation at 95°C for 10 min followed by 45 cycles of denaturation at 95°C for 15 s, annealing and extension at 60°C for 1 min. qRT-PCR reactions for quantification of transcript levels were conducted using 50 ng of first strand cDNA prepared from RNA samples isolated for the microarray analysis. The abundance of each transcript in an RNA sample was estimated from the corresponding gene's standard curve and normalized against RP S7 transcript abundance in the same RNA sample.

## Authors' contributions

AND carried out the cDNA library construction, microarray fabrication, data analysis, and drafted the manuscript. YSH performed the microarray experiment, data analysis, and helped draft the manuscript. MKK performed the qRT-PCR experiments and MEH annotated the ESTs. BWH constructed the microarray genechips and carried out microarray data acquisition. NFL sequenced the cDNA library and JRH maintained and provided mosquito samples throughout the project. PR assisted AND and YSH to draft the manuscript and reviewed it. FHC (P.I.) initiated and supervised the project. All authors read and agreed on the final version of this manuscript.

## Supplementary Material

Additional File 1 Supplementary Table S1 in a Microsoft Excel format where gene annotations for all 413 at least twofold differentially expressed gene products are given. Click here for file
